# The Common Accidents of Every-Day Life

**Published:** 1887-01-22

**Authors:** A. W. Hare

**Affiliations:** Senior Demonstrator of Surgery in the University of Edinburgh


### The Common Accidents of Every-day Life.

By A. W. Hare, M.B., F.R.C.S.E.
Senior Demonstrator of Surgery in the University of Edinburgh.

[Dr. Hare will be glad to receive instances of common accidents, and to answer inquiries addressed to him, care of the Editor of The Hospital.]

### Chapter I.—Early Misadventures.

Common accidents are, from the nature of the case, mostly commonplace affairs, and in their treatment they call for the employment chiefly of common sense. But common sense itself is at a loss, unless it be applied in conjunction with a knowledge of what immediate and ulterior dangers are to be avoided in individual cases. This knowledge, to be available for practical purposes in an emergency, must be absolutely accurate in quality, even though it be very limited in extent. Each period of human life has vital characteristics peculiarly its own, each is also attended by a train of possible mishaps special to itself. Both these aspects of each stage of existence must be shortly stated and clearly comprehended before the Common Accidents of Every-day Life can be dealt with satisfactorily.

From the commencement of human life to its end there are constantly slight variations in the total amount of vitality possessed by the individual. In respect to vital power, a human life may be represented by a curved line, starting from the level and gradually ascending till it reaches its maximum height; after which it gradually descends once more to the level, where it terminates. The two ends of this line represent, respectively, birth and death; the ascending part of the curve, the process of development and increase of vital power to a maximum; the descending part, the decay which naturally follows. This illustrates the fact that decay is as natural and healthy at one period of life as is growth at another; it also shows that an injury at one period of life, where vitality has not yet risen high, or has already partially diminished, will produce more disastrous effects than the same amount of injury at a time when vitality is at a higher level. Up to the prime of life there are constant additions to the sum-total of vitality; then comes a period of maintenance for a longer or shorter time, and this in turn gives place to a period of decay, ending sooner or later in dissolution:—

> "And so from hour to hour, we ripe and ripe;
> And then from hour to hour, we rot and rot:
> And thereby hangs a tale."

For convenience of description, different names have been universally assigned to the several stages of life, which will be made use of here. Infancy, childhood, boyhood, youth, manhood, and old age are terms which convey very definite ideas of the bodily and mental conditions peculiar to each period; each expresses facts in regard to the several stages of development, much more distinctly and accurately than could an artificial division of life into decades, or any other method of computation. Let us then discuss,—

> "First the *infant*, mewling and puking in its nurse's arms."

It will at once appear that the most striking feature of infancy is the very small amount of vitality already spoken of, constituting a state of weakness and unprotectedness that renders this period the most critical of all. As a fact, far more lives are lost during this interval than in any other, although it is the shortest of the periods of life as above defined. A striking illustration of this is found in the "Carlisle Tables," a series of calculations respecting the probable duration of life at various ages. The tables chronicle the birth of 10,000 children, and show that at the end of

| | | | |
|---|---|---|---|
| 1 year | ... ... ... ... | 8461 | survive. |
| 2 years | ... ... ... ... | 7779 | " |
| * | * * * * | * | |
| * | * * * * | * | |
| 41 years | ... ... ... ... | 5009 | " |
| 104 years | ... ... ... ... | 1 | " |

The second line shows that in the first two years of life, which constitute the stage of infancy, more than one-fifth of the total number born, die from one cause or another. How much of this enormous infantile mortality is preventible does not here immediately concern us. Suffice it to say, that it occurs chiefly in the less-educated classes, amongst whom the spread of sanitary knowledge may be expected to work beneficially in this respect. It illustrates how little resisting-power is present in infancy, and shews that even slight injuries must not be considered trifling during that period. Given, then, relatively low vitality, are there other conditions present in Infancy which render it liable to special accidents? To answer this, we must note the chief functions of infant life, and see if they are accompanied by any danger. Compared with the adult, the human infant has very few and very simple functions. In it all the complex mental processes of the adult are as yet in abeyance. Instead of intricately combined manual or bodily movements, it displays chiefly irregular and purposeless muscular activity. Purposeless, that is as regards any distinct *will* of its own, but of great importance as the outcome of an *instinct to move*, and thus to promote the nutrition of its muscles. In a word, the one great function of infancy is Bodily Nutrition, and an infant may be defined as a *purely assimilative human organism.* This function involves two stages: the receiving of regular supplies of food at suitable intervals; and periods of quiet for its proper digestion and absorption. Both stages are well seen in any healthy infant; it spends all its time in receiving or in digesting food; and, since its vital energies are almost entirely devoted to carrying on Nutrition, but little development of strength takes place in other directions, where it might have a protecting action. Consequently, the infant is exposed to two sources of danger: firstly, the risks that it may run in consequence of its non-discriminating instinct to swallow; secondly, in consequence of its complete helplessness, it may suffer from the carelessness of its natural or deputed protectors.

I. Dangers Connected with Swallowing.

(*a.*) **Causes of Choking in Infants.** The lodgment of a foreign body or portion of solid food in the throat; giving too much liquid at a time, or any liquid too hot or too pungent to be swallowed; the presence in the throat of thick viscid phlegm, etc., etc. Some of these causes are of course preventible, and, with proper care, no infant should suffer from them. No breakable toy, nor any that could possibly be put whole into the mouth should be allowed, nor any that could be deleterious if sucked and bitten, as the instinct of the infant will certainly lead it to this action. Hard, solid food should never be given, and liquids only in small quantities at a time, of moderate heat and suitable composition.

**The Symptoms of Choking.** These are gurgling noises in the throat; spasmodic attempts to draw in breath, preceded by ineffectual coughing; contortions of the body and limbs, the hand at times thrust into the mouth, as though to remove an obstruction; the face first red, then getting damask, then purple, lastly very dark and livid; the eyes prominent, fixed and staring. Unless relief can be at once afforded, this stage passes on rapidly to death.

**The Treatment of Choking.** Choking results from a closure of the entrance of the air-passages leading to the lungs, either by a foreign body which mechanically blocks up the entrance, or by a spasm of the muscles of the throat, caused by an irritating material lying in contact with very sensitive surfaces. The object of treatment is to get rid of the source of the symptoms as quickly as possible. To do this, first place the infant in the best position for relief; from lying on its back, which is the worst possible position, alter its posture to one of leaning forward; supporting the front of its chest on the palm of the right hand; the left hand on the upper part of its back. If a liquid be the cause of the choking, it is easily expelled in this posture, and the child recovers. Should the symptoms continue, pat the back of the little patient gently three or four times, to dislodge any solid particle which may be present, and to cause its expulsion; and, in doing this, do not exceed the limits of extreme gentleness, or the result may be an increase of the urgency of the symptoms, the lungs having been still further emptied of much-needed air. If not immediately relieved, lose no time in passing the fore-finger of the right hand into the patient's mouth, push it as far back as the root of the tongue, and feel whether there is any foreign body obstructing the throat; should one be felt, it must be at once removed by hooking the finger over the top of it and withdrawing the hand. Or, two fingers may be introduced, and the object grasped between them and removed, unless it is firmly fixed, when it may be dislodged by using sugar-tongs for the same purpose. If nothing be felt by the exploring finger, it does not prove that no foreign body is present, for it may be out of reach; to get it dislodged, if present, the upper surface of the back of the tongue must be tickled; this occasions violent attempts at vomiting, and any foreign matter is almost certainly expelled. Should this line of treatment give no relief, a surgeon must be sent for with the utmost haste. If there is a prospect of his immediate arrival, the neck should be uncovered, as his first duty will be to open the wind-pipe in that region; at the same time the body and limbs must be kept warm, and in the horizontal position; a large sponge, and bowls of hot and cold water should also be provided, as the surgeon will probably use their contents, alternately dashed upon the chest, to excite respiratory movements after the wind-pipe has been opened.

(*To be continued.*)

